# TGF-β1-Mediated Differentiation of Fibroblasts Is Associated with Increased Mitochondrial Content and Cellular Respiration

**DOI:** 10.1371/journal.pone.0123046

**Published:** 2015-04-07

**Authors:** Ulugbek Negmadjanov, Zarko Godic, Farhan Rizvi, Larisa Emelyanova, Gracious Ross, John Richards, Ekhson L. Holmuhamedov, Arshad Jahangir

**Affiliations:** 1 Sheikh Khalifa bin Hamad Al Thani Center for Integrative Research on Cardiovascular Aging, Aurora Research Institute, Aurora Health Care, Milwaukee, Wisconsin, 53215, United States of America; 2 Laboratory of Immunology, Aurora Health Care, Milwaukee, Wisconsin, 53215, United States of America; 3 Aurora Cardiovascular Services, Aurora Health Care, Milwaukee, Wisconsin, 53215, United States of America; RIKEN Advanced Science Institute, JAPAN

## Abstract

**Objectivs:**

Cytokine-dependent activation of fibroblasts to myofibroblasts, a key event in fibrosis, is accompanied by phenotypic changes with increased secretory and contractile properties dependent on increased energy utilization, yet changes in the energetic profile of these cells are not fully described. We hypothesize that the TGF-β1-mediated transformation of myofibroblasts is associated with an increase in mitochondrial content and function when compared to naive fibroblasts.

**Methods:**

Cultured NIH/3T3 mouse fibroblasts treated with TGF-β1, a profibrotic cytokine, or vehicle were assessed for transformation to myofibroblasts (appearance of α-smooth muscle actin [α-SMA] stress fibers) and associated changes in mitochondrial content and functions using laser confocal microscopy, Seahorse respirometry, multi-well plate reader and biochemical protocols. Expression of mitochondrial-specific proteins was determined using western blotting, and the mitochondrial DNA quantified using Mitochondrial DNA isolation kit.

**Results:**

Treatment with TGF-β1 (5 ng/mL) induced transformation of naive fibroblasts into myofibroblasts with a threefold increase in the expression of α-SMA (6.85 ± 0.27 RU) compared to cells not treated with TGF-β1 (2.52 ± 0.11 RU). TGF-β1 exposure increased the number of mitochondria in the cells, as monitored by membrane potential sensitive dye tetramethylrhodamine, and expression of mitochondria-specific proteins; voltage-dependent anion channels (0.54 ± 0.05 vs. 0.23 ± 0.05 RU) and adenine nucleotide transporter (0.61 ± 0.11 vs. 0.22 ± 0.05 RU), as well as mitochondrial DNA content (530 ± 12 μg DNA/10^6^ cells vs. 307 ± 9 μg DNA/10^6^ cells in control). TGF-β1 treatment was associated with an increase in mitochondrial function with a twofold increase in baseline oxygen consumption rate (2.25 ± 0.03 vs. 1.13 ± 0.1 nmol O_2_/min/10^6^ cells) and FCCP-induced mitochondrial respiration (2.87 ± 0.03 vs. 1.46 ± 0.15 nmol O_2_/min/10^6^ cells).

**Conclusions:**

TGF-β1 induced differentiation of fibroblasts is accompanied by energetic remodeling of myofibroblasts with an increase in mitochondrial respiration and mitochondrial content.

## Introduction

Fibroblasts are the major cells involved in extracellular matrix remodeling and the repair processes following injury through cytokine-dependent transformation into myofibroblasts [1–4]. Differentiation of fibroblasts into myofibroblasts for active repair of damaged tissue is accompanied by major changes in cell phenotype with conversion of non-excitable precursors into excitable myofibroblasts, cells with increased contractility and higher synthetic and secretory capabilities [5–9], processes that increase cellular energy demands [10]. Although phenotypic changes with fibroblast differentiation are well characterized, little information is available about mitochondrial remodeling associated with fibroblast differentiation. The aim of this study was to evaluate the changes in mitochondrial content and respiration of fibroblasts treated with vehicle or transforming growth factor-β1 (TGF-β1), a profibrotic cytokine known to activate fibroblasts into myofibroblasts [1,2].

## Materials and Methods

### Propagation and storage of NIH/3T3 fibroblasts

Murine NIH/3T3 cells were purchased from American Type Culture Collection (Manassas, VA) and propagated in high-glucose ATCC-DMEM media (ATCC, USA) supplemented with 10% newborn bovine calf serum (BCS, GIBCO, USA) and 1% penicillin/streptomycin (GIBCO, USA) in a cell culture incubator in a 5% carbon dioxide (CO_2_)/95% air environment. Cultured cells at 50–60% confluence were detached using 0.05% trypsin/EDTA; transferred into freezing media composed of high glucose ATCC-DMEM supplemented with 1% penicillin/streptomycin, 15% bovine calf serum and 10% DMSO; and stored in liquid nitrogen. Cells were plated at the initial density of 5,000 cells/cm^2^ and allowed to attach overnight in a humidified cell culture incubator at 37°C in 5% CO_2_/95% air before proceeding with treatments. All experiments were performed in accordance with Aurora Health Care institutional policies for research.

### TGF-β1 treatment and differentiation

TGF-β1-dependent differentiation of NIH/3T3 cells was conducted as previously described [2,9,11–14]. Briefly, NIH/3T3 cells were seeded at 5,000 cells/cm^2^ and incubated overnight (16–20 hours) to allow attachment in a monolayer, and following 24 hours of incubation in 2.5% serum media, cells were treated with either 5 ng/mL of recombinant TGF-β1 (Sigma, USA) or vehicle. After 48 hours, cells were rinsed with Dulbecco’s Phosphate-Buffered Saline (DPBS), detached using 0.05% trypsin/EDTA and used for experiments.

### Immunocytochemistry

Identification of naive and differentiated fibroblasts was performed using immunocytochemistry with visualization of vimentin and α-smooth muscle actin (α-SMA) marker proteins of naive and differentiated fibroblasts, respectively, as per Abcam protocol <http://www.abcam.com/index.html?pageconfig=resource&rid=11459>. Briefly, cells were fixed with 4% paraformaldehyde (Sigma, USA), treated with 90% methanol and incubated for 1 hour in blotting buffer DPBS supplemented with 0.5% bovine serum albumin (BSA, Sigma, USA). The fixed and permeabilized cells were then incubated (1 hour) with the mixture of primary goat polyclonal anti-mouse vimentin antibodies (Abcam, USA) and rabbit polyclonal anti-mouse α-SMA antibodies (Abcam, USA). To visualize vimentin and α-SMA cells, they were incubated (1 hour) in fluorescently labeled secondary donkey anti-goat AlexaFluor488 (H+L) (Life Technology, USA) and donkey anti-rabbit AlexaFluor594 (H+L) (Life Technology, USA) antibodies, respectively, as per manufacturer recommendations. The labeled cells were then washed in DPBS buffer, transferred into FluoroShield mounting medium, supplemented with 4′,6-Diamidino-2-phenylindole dihydrochloride (Sigma-Aldrich, USA) and imaged using an Olympus IX71 inverted microspore equipped with an Olympus DP72 CCD camera (Olympus, USA) and/or using an Olympus FL 1200 MPE laser confocal microscopy system. Quantification of red and green fluorescence was performed using Image*J*, free access National Institutes of Health (NIH) software (http://rsb.info.nih.gov/ij/), as described [15].

### Mitochondrial imaging

NIH/3T3 fibroblasts were seeded on glass-bottom MatTek dishes (MatTek Corp., USA) coated with rat tail type I collagen, as described [16,17]. Adhered cells were treated with vehicle or TGF-β1 (5 ng /mL) for 48 hours, as described above. For life cell imaging of cell nuclei and intracellular mitochondria, cells were loaded with cell-permeable nuclear-selective fluorescent dye Hoechst 33342 (Molecular Probes, USA), and red fluorescing mitochondrial membrane potential sensitive dye tetramethylrhodamine (TMRM) (Molecular Probes, USA). The fluorescent images of stained cells were obtained using an Olympus FV1200 MPE laser confocal microscope (Olympus, USA) equipped with a long working distance dry UPLSAPO 40X/0.95 lens with corrective collar and appropriate excitation laser lines with corresponding dichroic cubes (Hoechst 33342; excitation 405 nm/emission 470 and TMRM; excitation 569 nm/emission 590 nm).

### Western blotting

The samples were separated using NuPAGE Novex 4–12% Bis-Tris 1 mm-thick mini-gels (Life Technologies, USA). Briefly, gels loaded with 20 μg proteins/lane and run at 110 V for about 2 hours at room temperature were transferred onto a polyvinylidene difluoride membrane using the iBlot dry blotting system (Life Technologies, USA). The polyvinylidene difluoride membranes were incubated on belly-dancer in blotting buffer—tris-buffered saline (TBS) containing 3% non-fat dry milk and 0.1% Tween-20—for 1 hour at room temperature. Further, membranes were incubated with the mixture of antibodies against α-SMA (1:1,000 dilutions), vimentin (1:5,000 dilution) and GlycerAldehyde 3-Phosphate Dehydrogenase (GAPDH) (1:1000 dilution) dissolved in blotting buffer. Following incubation with primary antibodies, the membranes were washed with 1x TBS with 0.1% Tween-20 three times at 5-minute intervals before incubation for 1 hour at room temperature with horse radish peroxidase conjugated donkey anti-mouse IgG (Santa Cruz, USA) and goat anti-rabbit polyclonal antibody (Abcam, USA) in blotting buffer. The membranes were washed with 1x TBS with 0.1% Tween-20 three times at 5-minute intervals and twice in 1x TBS without Tween-20, to avoid an inhibitory effect of Tween-20 on the detection method. The bands on the membrane were visualized using Super Signal West Pico Chemiluminescent Substrate (Thermo Fisher, USA) and monitored using Molecular Imaging Systems (UltraLum, Claremont, CA) and Ultra Quant v6.0 software (http://ultraquant.software.informer.com/6.0/). The density of the obtained protein bands was analyzed using NIH Image*J* software and bands of interest were normalized to the density of the respective GAPDH band.

### Quantification of mitochondrial DNA in naive and differentiated NIH/3T3 cells

Mitochondrial DNA was isolated from cultured NIH/3T3 cells using a mitochondrial DNA isolation kit (BioVision, Milpitas, Calif., USA) as per the manufacturer’s guidelines [18]. Briefly, NIH/3T3 fibroblasts grown in the absence and presence of TGF-β1 (5 ng/mL) were harvested after 48 hours and mitochondrial DNA was extracted from the cell suspension as recommended by the manufacturer (BioVision, USA), quantified using Tecan’s Nano Quant Infinite 200 plate reader (Tecan, USA) and expressed as micrograms (μg) of mitochondrial DNA per million cells.

### Seahorse respirometry

Cellular respiration was quantified using the Seahorse Extracellular Flux Analyzer XF-96 (Seahorse Biosciences, USA) as described [16,17,19,20]. Each 96-well Seahorse cell culture plate was pre-coated with rat tail type I collagen (Sigma, USA) as described [16,17]. Naive and differentiated NIH/3T3 cells were detached using 0.05% trypsin/EDTA, counted and plated at 20,000 cells-per-well density. Cells were allowed to attach for 16–20 hours. The initial incubation medium was replaced with Seahorse incubation medium and then the plate was transferred into the XF-96 analyzer for calibration and measurement of oxygen consumption rate (OCR) [17,19,20]. The duration of each step in the measurement cycle was adjusted to: Mix—1 minute, Wait—2 minutes, Measure—3 minutes, as we previously described [16,17]. Respiration of cells was quantified as OCR at baseline and following treatment of each well with appropriate mitochondrial modulators such as oligomycin (1μg/mL), FCCP (0.1 μM) and/or antimycin A (1 μg/mL).

### Propidium iodide-based quantification of adhered cells in 96-well Seahorse plate

**Fig 1:**
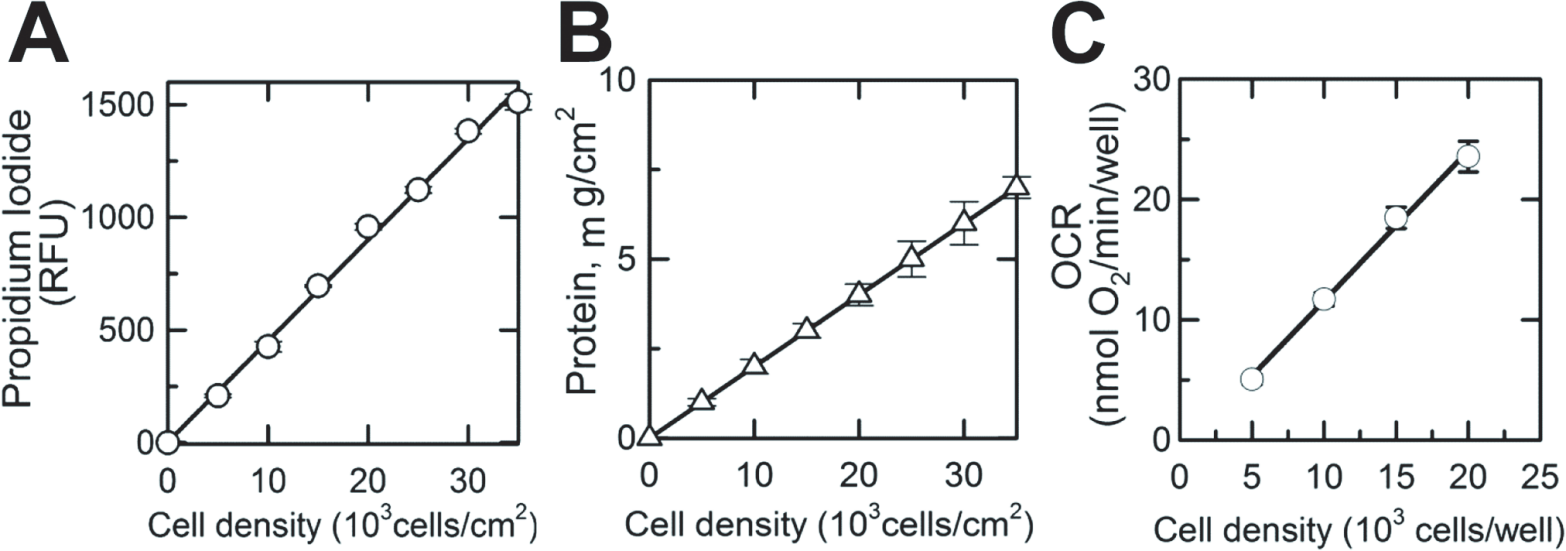
Propidium Iodide-based quantification of the number of viable cells in a 96-well Seahorse microplate. **A**, Propidium Iodide fluorescence (Relative Fluorescence Unit) measured from individual wells of a XF 96-well microplate (excitation 535 nm; emission 617 nm) versus the number of cells plated into the well (cells/cm^2^). **B**, Protein content measured in each well versus cell density in these wells (number of cells plated into well, cells/cm^2^). **C**, Linear relationship between cell density (number of cells plated into well, cells/cm^2^) and OCR (oxygen consumption rate in corresponding wells). Shown are averages of at least three independent measurements ± standard error of mean.

Quantification of cell number in each well of the 96-well plate was performed using propidium iodide, a nuclear-specific fluorescent-dye-based assay. Briefly, the incubation media from each of the 96 wells was removed after the completion of cell respiration measurement, and attached cells were rinsed with 200 μL of warm DPBS, 200 μL of warm DPBS supplemented with 50 μM of digitonin and then 3 μg/mL of propidium iodide. The plate was then incubated in a humidified cell culture incubator at 37°C for 30 minutes. The intensity of the fluorescence of propidium iodide (excitation 535 nm; emission 617 nm) bound to the nuclei of digitonin-permeabilized cells was measured using a Tecan Infinity 200PRO multi-well plate reader (Tecan Ltd., Switzerland). As illustrated in Fig 1, a linear relationship was observed between cell density (within the range of 0–35,000 cells/cm^2^), propidium iodide fluorescence (Fig 1A) and total cell protein (Fig 1B, DC Bio-Rad Protein Assay USA). The respiration of attached cells in individual wells (OCR/well) linearly increased with the cell density, allowing normalization of the OCR in each well (Fig 1C).

### ATP/ADP ratio in naive and differentiated NIH/3T3 cells

The ATP/ADP ratio in untreated- and TGF-β1-treated cells was measured using an ADP/ATP ratio luminescent kit (Sigma-Aldrich, USA) according to manufacturer’s guidelines. Luminescence was measured using the Tecan Infinite M200 PRO plate reader (Tecan USA). Briefly, NIH/3T3 fibroblasts grown in the absence and presence of TGF-β 1 (5 ng/mL) for 48 hours were harvested and plated at a density of 50,000 cells/cm^2^ in a 96-well plate coated with rat tail type I collagen (15 μg/cm^2^). Following overnight incubation, the ratio of ATP/ADP was determined.

### Drugs

All chemicals were purchased from Sigma-Aldrich Chemicals (St. Louis, MO) unless indicated differently. Tetramethylrhodamine methyl ester and Hoechst 33342 were from Life Technologies, USA. Primary goat anti-mouse polyclonal antibodies to vimentin (Abcam, USA) were paired with secondary AlexaFluor488 donkey anti-goat IgG (H+L), (Life Technologies, USA), and primary rabbit anti-mouse polyclonal antibodies to αSMA (Abcam, USA) were paired with secondary donkey anti-rabbit IgG (H+L) conjugated with AlexaFluor594 (Life Technologies, USA).

### Statistical analysis

Data were expressed as means ± standard error of mean, and “n” represents the number of repeats. Comparison between groups was made using Student t-test analysis; p <0.05 was considered statistically significant.

## Results

### TGF-β1 treatment enhances expression of α-SMA in fibroblasts

**Fig 2:**
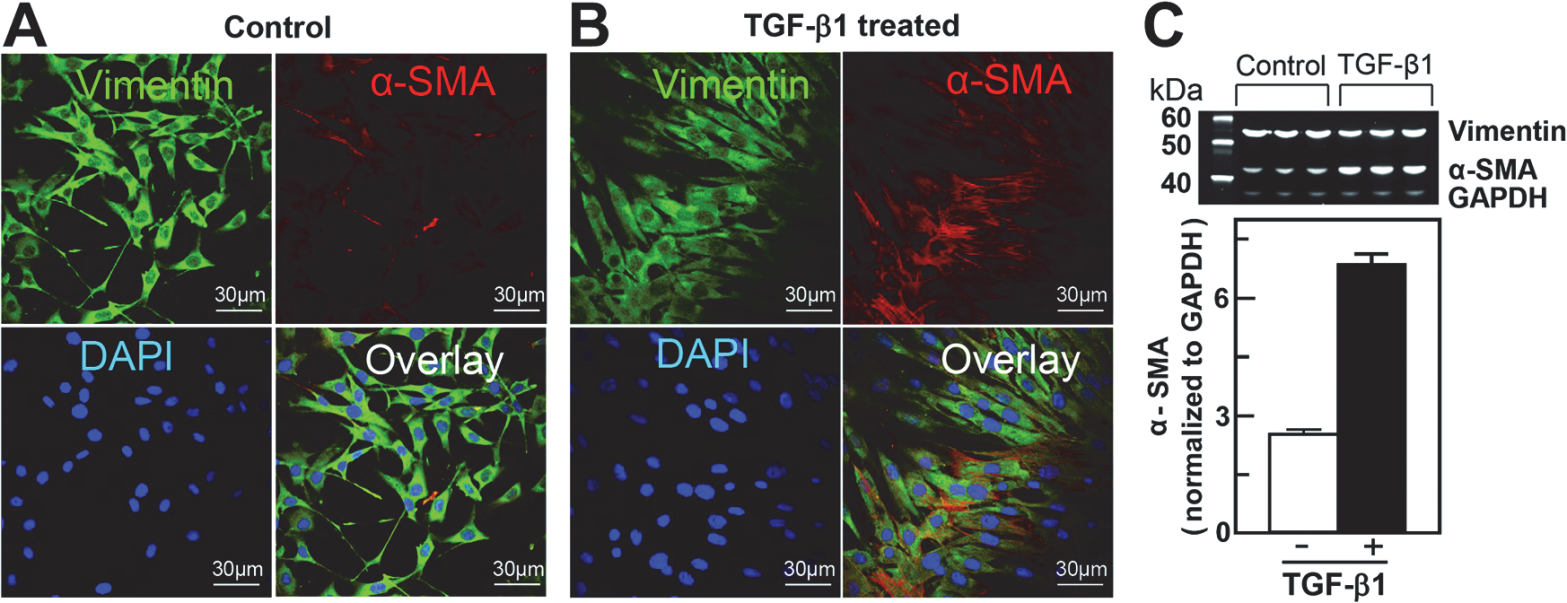
TGF-β1-mediated differentiation of NIH/3T3 fibroblasts into myofibroblasts. **A and B**, Representative fluorescent images of naive (control) and differentiated (TGF-β1-treated) cells. Cell nuclei are stained blue (1 μg/mL in mounting media); intracellular vimentin, a characteristic protein of fibroblasts, and α-smooth muscle actin (α-SMA), a marker of myofibroblasts (green and red, respectively), were labeled using immunocytochemistry as described in the Materials and Methods section. Superimposed images of naive and differentiated NIH/3T3 cells (overlay) demonstrate increased expression of α-SMA following TGF-β1 treatment. **C**, Relative increase in expression of α-SMA in NIH/3T3 cells following differentiation (average of at least 3 experiments). Densitometry of western blot bands was performed using Image*J* software as described in the Materials and Methods sections.

Exposure of cultured NIH/3T3 cells to exogenous TGF-β1 (5 ng/mL, 48 hours) promoted transformation of naive fibroblasts into differentiated myofibroblasts (Fig 2A and 2B). Fluorescent images of vimentin, a marker of naive fibroblasts (Fig 2, green fluorescence), and α-SMA, a marker of differentiated myofibroblasts (Fig 2, red fluorescence), were taken using an Olympus FV1200 MPE multicolor laser scanning confocal microscope (Olympus Inc., USA). Cellular nuclei were labeled with Hoechst 33342, a cell-permeable DNA-specific fluorescent dye (Fig 2, blue fluorescence). TGF-β1-untreated cells (Fig 2A) manifested typical morphology of cultured fibroblasts with dendritic shape and projections extending from the main cell body expressing vimentin (Fig 2A, green fluorescence) with minimum expression of α-SMA (Fig 2A, red fluorescence). TGF-β1-treated cells, on the other hand, assumed a more flattened, spread out shape with a larger cell size and nucleus and expressed a fourfold-higher level of α-SMA stress fibers crossing the cell cytoplasm within the vimentin-positive cells (Fig 2B), indicating a fourfold greater transformation of the fibroblasts into myofibroblasts compared to the TGF-β1-untreated cells (Fig 2). A quantitative estimate for α-SMA expression in naive and differentiated NIH/3T3 cells (Fig 2C) was assessed using western blotting and Image*J* software. No significant change in the vimentin to GAPDH ratio was observed between the naive (0.87 ± 0.08; n = 3) and differentiated (0.63 ± 0.05; n = 3) NIH/3T3 cells, compared to a 2.5-fold change in the level of α-SMA expression in TGF-β1-treated NIH/3T3 cells (Fig 2C, n = 3).

### TGF-β1 treatment increases expression of mitochondria-specific proteins in NIH/3T3 fibroblasts

**Fig 3:**
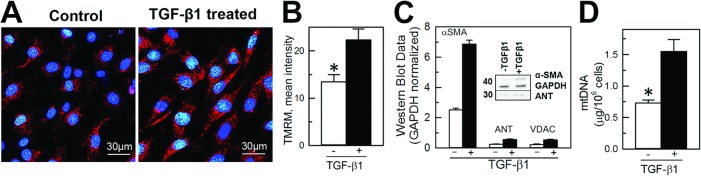
TGF-β1 treatment increased the content of mitochondria and expression of mitochondria-specific proteins and mitochondrial DNA in differentiated NIH/3T3 fibroblasts. **A**, Confocal images of NIH/3T3 cells loaded with nuclear (Hoechst 33342, blue) and mitochondria-specific (tetramethylrhodamine, red) fluorescent dyes. **B,** Quantification of the mean intensity of TMRM in naive and TGF-β1-treated cells using Image*J*. **C,** Quantification of α-SMA (marker of myofibroblasts), adenine nucleotide transporter (ANT) and voltage-dependent anion channels (VDAC) expression in naive (TGF-β1- untreated) and differentiated (TGF-β1-treated) NIH/3T3 cells. Densities of western blot bands were normalized to glyceraldehyde-6-phosphate dehydrogenase (GAPDH), a house-keeping protein. **D**, Quantification of mitochondrial DNA in naive and TGF-β1-treated cells. Shown are averages of at least three independent measurements ± standard error of mean, and the asterisk shows significance, p<0.05. E, Quantification of ATP/ADP ratio in naive and TGF-β1 treated cells (n = 3, p<0.05).

To determine if fibroblast transformation into myofibroblasts is associated with changes in mitochondrial content, a multi-parametric approach was used with visualization of mitochondria within TGF-β1- untreated (vimentin positive, α-SMA negative cells) and-treated cells (vimentin and α-SMA positive cells) and quantification of mitochondrial-specific proteins and mitochondrial DNA content. Naive and differentiated NIH/3T3 cells were loaded with TMRM, a mitochondrial membrane potential sensitive fluorescent dye, and the presence of active polarized mitochondria was determined by punctate red fluorescence (TMRM) around the nucleus (Hoechst 33342) using laser confocal microscopy (Fig 3A). Qualitatively, mitochondria appeared to be present in greater numbers in TGF-β1-treated cells compared to untreated cells (Fig 3A). Quantification of the mean intensity of TMRM in naive and TGF-β1-treated cells demonstrated on average almost two-fold increase of TMRM fluorescence from 13.4 ± 1.6 to 22.3 ± 2.3 RFU (Fig 3B). Similar to an increase in α-SMA expression, western blot of the mitochondrial-specific proteins ANT (adenine nucleotide transporter) and VDAC (voltage-dependent anion channels) expression normalized to the housekeeping protein GAPDH was also significantly higher in the TGF-β1-treated cells (Fig 3B, insert). The expression of α-SMA in untreated versus treated cells increased from 0.81 ± 0.13 RU to 2.32 ± 0.17 RU, the expression of ANT from 0.22 ± 0.05 RU to 0.61 ± 0.11 RU and total VDAC from 0.23 ± 0.05 RU to 0.54 ± 0.05 RU (Fig 3B). In addition, the overall content of mitochondrial DNA in untreated and TGF-β1-treated cells significantly increased from 0.69 ± 0.05 μg DNA/10^6^ cells to 1.39 ± 0.18 μg DNA/10^6^ cells in differentiated myofibroblasts (n = 3; p<0.05, Fig 3C).

### TGF-β1-induced differentiation of NIH/T3 cells results in enhanced mitochondrial respiration, increased ATP/ADP ratio, and glucose and pyruvate oxidation

**Fig 4:**
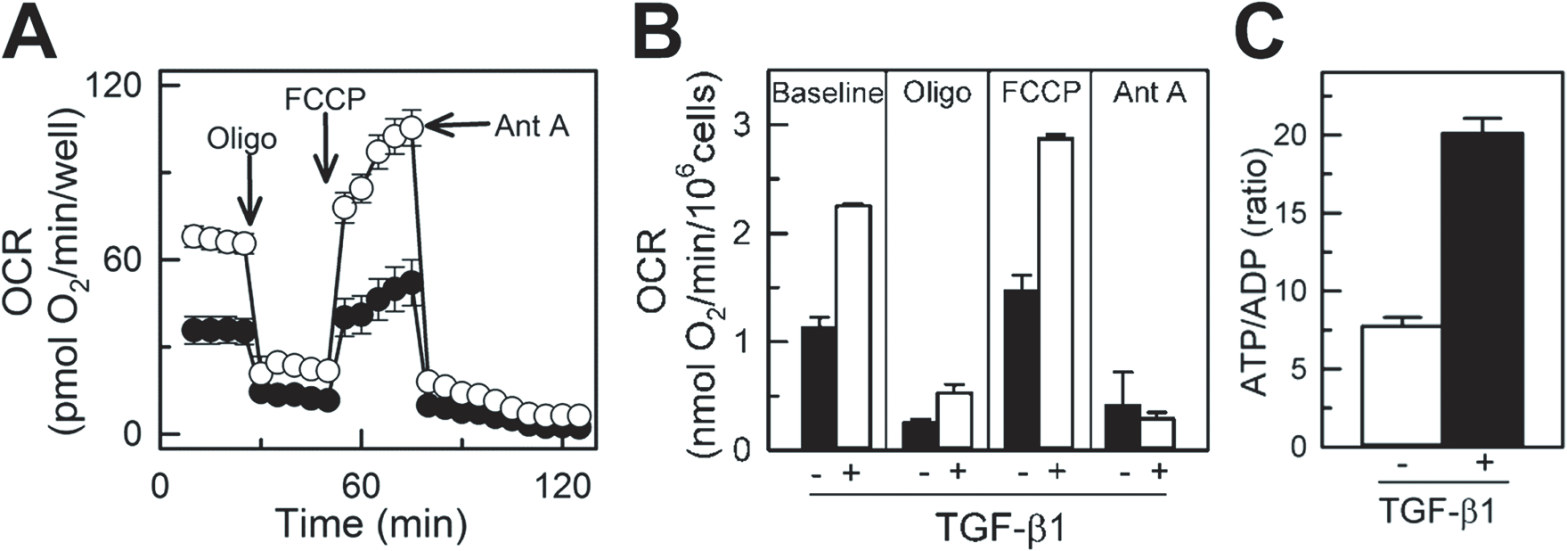
TGF-β1-mediated enhancement of mitochondrial respiration in NIH/3T3 cells. **A**, Endogenous oxygen consumption rate (OCR) of naive (TGF-β1-untreated) and differentiated (TGF-β1-treated) NIH/3T3 cells measured using Seahorse XF-96. Basal respiration was inhibited with oligomycin (inhibitor of oxidative phosphorylation), followed by treatment with FCCP (uncoupler of mitochondrial oxidative phosphorylation) and antimycin A (inhibitor of mitochondrial respiratory chain). **B**, Quantification of OCR in naive and TGF-β1-treated NIH/3T3 cells at baseline and following treatment with oligomycin, FCCP and antimycin A. Shown are averages of at least three independent measurements ± standard error of mean. White circles and bars for naive, TGF-β1-untreated cells and black circles and bars for TGF-β1-treated cells.

Respiration of immobilized naive and differentiated NIH/3T3 cells was measured using Seahorse respirometry. The respiratory rate of cells in the 96-well plate linearly increased with increased density of cells from 5,000 cells/well to 20,000 cells/well (Fig 1C), demonstrating that Seahorse respirometry within the given range of cell density is suitable for assessing respiration of plated cells. Representative time-dependent changes in OCR in control and TGF-β1-treated cells are shown in Fig 4A. Each time-point is shown as an average OCR from at least 4 wells from 3 independent experiments. Baseline respirations of naive and differentiated NIH/3T3 cells measured in standard incubation media (Seahorse Biosciences XF Assay medium, Cat # 102352–000) were 1.13 ± 0.09 and 2.25 ± 0.02 pmol O_2_/min/10^6^ cells, respectively (Fig 4B), which was suppressed by oligomycin, a specific inhibitor of mitochondrial ATP synthase and oxidative phosphorylation. The maximal respiration of naive and TGF-β1-treated cells determined after uncoupling of mitochondria with FCCP was 1.46 ± 0.15 and 2.87 ± 0.04 pmol O_2_/min/10^6^ cells, respectively. Non-mitochondrial cellular respiration was assessed in cells following treatment with antimycin A, a selective mitochondrial respiratory chain inhibitor. In naive cells this respiration dropped from the maximal rate of oxygen consumption (FCCP) to 0.47 ± 0.03 pmol O_2_/min/10^6^ cells, a 3.5-fold decrease as compared with TGF-β1-treated cells that dropped from the maximal (2.87 ± 0.04 pmol O_2_/min/10^6^ cells) to 0.29 ± 0.02 pmol O_2_/min/10^6^ cells, a 10-fold decline (Fig 4B and 4C). Increased mitochondrial respiration in TGF-β1-treated NIH/3T3 cells was paralleled by a 3-fold increase in ATP/ADP ratio (from 7.75 ± 0.56 in naive to 20.12 ± 0.95 in TGF-β1-treated cells), indicating enhanced mitochondrial ATP turnover and cell metabolism in TGF-β1-treated cells (Fig 4D). The rate of glucose oxidation increased from 192.8 ± 23 nmol/h/10^6^ cells in naive cells to 236.9 ± 37 nmol/h/10^6^ cells in TGF-β1-treated NIH/3T3 cells. Increased utilization of glucose by TGF-β1-treated cells was reflected by decreased accumulation of pyruvate, as seen from the increased ratio of lactate/pyruvate from 3.1 in undifferentiated fibroblasts to 5.0 in differentiated myofibroblasts.

## Discussion

The main finding of our study is that TGF-β1 transformation of fibroblasts to myofibroblasts is accompanied by an increase in functioning mitochondrial content, as reflected by a higher number of polarized mitochondria on confocal microscopy, and increase in mitochondrial-specific protein and mitochondrial DNA levels, and this was associated with a higher capacity of these transformed cells to mitochondrial respiration and ATP generation. This study, to our knowledge, is the first to describe mitochondrial remodeling of fibroblasts undergoing cytokine-mediated differentiation to myofibroblasts, a phenotype that requires higher energy due to the increase in the contractile and secretory functions of these cells, critical for wound healing.

Fibroblasts are a critical component of the repair mechanism, becoming the dominant cell type within a wound after injury and producing a number of chemotactic signals attracting other cells and additional fibroblasts, directing their migration into the wound. TGF-β1 and other cytokines facilitate their activation into myofibroblasts that synthesize and secrete extracellular matrix proteins and also promote contraction of the scar [21–23]. All these complex processes in wound healing are highly energy dependent, facilitated by a higher ATP level [1,2,24–27] and, thus, require a change in the energetics of the cell to meet the higher energy demands associated with the healing process [25,26]. Although mitochondria have been recognized as an essential organelle involved in cell injury and repair [28,29], contributing to the increased energy demands of the healing tissue, very little information is available [30–32] about the changes in mitochondrial content or function with activation of fibroblasts to myofibroblasts. Since mitochondria produce 17 times more ATP during oxidative phosphorylation than anaerobic glycolysis [33,34] we hypothesize that mitochondria will undergo remodeling with transformation of fibroblasts to myofibroblasts to meet the increased energy demands of these cells in wound healing and tissue repair [35,36,37].

TGF-β1 treatment of NIH/3T3 fibroblasts resulted in increased transformation of these cells into activated myofibroblasts, documented by increased expression of α-SMA (Fig 2). TGF-β1-induced transformation of fibroblasts to myofibroblasts and associated remodeling of mitochondria was demonstrated by increased intensity of TMRM mitochondrial membrane potential sensitive dye, and further confirmed by increased expression of specific mitochondrial integral proteins (adenine nucleotide transporter and voltage-dependent anion channels) and mitochondrial DNA content (Fig 3). This was paralleled by an increased rate of oxygen consumption among cells expressing a high level of α-SMA (Fig 4). This increase in oxygen consumption was due to enhanced oxidative phosphorylation capacity as confirmed by sensitivity to oligomycin and antimycin, two specific mitochondrial inhibitors. Increased mitochondrial metabolism in myofibroblasts was further confirmed by increased ATP/ADP (Fig 4C) ratio and increased lactate/pyruvate ratio. Our observation is in line with previous reports demonstrating that interventions that improve and accelerate wound healing are associated with changes in cell shape and morphology from what has been described as resting state [38,39] to active state with an increased ability to release ATP [36,40] and increase in mitochondrial-specific protein (cytochrome c-oxidase), energy charge and ATP/ADP ratio [36]. Although the authors did not specifically look at the transformation of fibroblasts to myofibroblasts, their description of changes in cell morphology and mitochondrial function are similar to what we describe and are suggestive of fibroblast to myofibroblast transformation with enhanced production of TGF-β1 and platelet-derived growth factors and fibroblast activation to myofibroblasts [21,36].

In summary, the current study, the first to assess the effect of TGF-β1-induced transformation of fibroblasts to myofibroblasts on changes in mitochondrial content and oxygen consumption rate, indicates a metabolic remodeling of the activated fibroblasts to meet the increased energetic demands associated with the enhanced secretory, synthetic and contractile function of myofibroblasts essential for normal wound healing processes [41,42]. Future studies on disease conditions that alter the normal healing process, such as diabetes mellitus [30,31] or ischemic tissue [41,42], defining the role of fibroblast/myofibroblast energetics in delayed or impaired wound healing and the impact of targeting mitochondria to improve energetics and accelerate repair after injury are warranted.
